# Synovial sarcoma of the spine: A report of three cases and review of the literature

**DOI:** 10.4103/2152-7806.76939

**Published:** 2011-02-21

**Authors:** Ross C. Puffer, David J. Daniels, Caterina Giannini, Mark A. Pichelmann, Peter S. Rose, Michelle J. Clarke

**Affiliations:** Mayo Clinic, 200 1^st^ St SW Rochester, MN 55905, Mayo Medical School, MN 55905, USA; 1 Department of Neurosurgery, Mayo Clinic, 200 1^st^ St SW Rochester, MN 55905, USA; 2 Department of Anatomic Pathology, Mayo Clinic, 200 1^st^ St SW Rochester, MN 55905, USA; 3 Department of Orthopedic Surgery, Mayo Clinic, 200 1^st^ St SW Rochester, MN 55905, USA

**Keywords:** Case series review, Spinal synovial sarcoma, Treatment, Negative margins

## Abstract

**Background:**

Synovial sarcoma (SS) is a rare sarcoma with distinct morphologic and genetic features, which, despite its name, does not arise from synovium. While most SSs (>80%) arise in the deep soft tissue of the extremities, up to 5% of these tumors are encountered in the body axis including the spine, mediastinum, retroperitoneum, and head/neck regions. Reports of SS located within the spinal axis have been rare to date.

**Materials and Methods:**

We searched the medical records at our institution and found three patients who were diagnosed and treated for SSs involving the spine. We also performed an exhaustive literature search using PubMed to identify all reported cases in the literature.

**Results:**

In this study, we report on three SS cases involving the spine. All three cases involved the paraspinal muscles and spinal nerve roots, with one case having a significant leptomeningeal involvement. In two cases, “smaller operations” were performed first because the lesions were thought to be benign, however, when the final pathology identified them as SSs, more radical procedures were performed. Additionally, we identified 14 cases of SSs involving the spine published in the literature and all cases are reviewed here.

**Conclusions:**

Due to limited numbers of cases, spine SS long-term outcomes are hard to quantify. The currently accepted standard of treatment for SSs starts with wide surgical excision with negative margins followed by chemotherapy and radiation. We summarize the available literature on spinal SSs and review the current treatment options available for these tumors.

## INTRODUCTION

Synovial sarcoma (SS) is a rare soft tissue tumor comprising 5–10% of soft tissue sarcomas and less than 1% of all malignancies.[6,7,16,30] SS affects mainly adolescents and young adults with a peak incidence in the third decade. Approximately, 30% of cases occur before the age of 20, and 90% before 50.[7,30] Despite its name, SS does not originate and/or differentiate toward synovium (less than 5% of cases arise from joints or bursa). Although SS can occur in any part of the body, more than 80% of tumors arise in the deep soft tissue of the extremities, especially around the knee. The etiology of SS is unknown but they appear to have an epithelial phenotype. Although it is known for being particularly aggressive, SS often grows slowly, forming a circumscribed, multinodular tumor without a capsule.[6] More than 90% of SS show a consistent, balanced reciprocal translocation t(X:18)(p11:q11) presumably relevant in its pathogenesis. This translocation involves the fusion of the SYT gene at 18q11 to either homologous genes SSX1 or SSX2 at Xp11.[6,9,14,25,30,33] This allows for several histopathological variants, including monophasic, biphasic and poorly differentiated forms.[6,14,33] Although there is no significant correlation among tumor location, metastases at time of diagnosis, age, sex, or the type of transcript, in patients with localized tumors, SYT-SSX2 fusion transcripts seem to predict significantly longer metastasis-free survival than SYT-SSX1 fusion transcripts.[14]

Surgical excision with wide, negative margins is the currently recommended treatment with adjuvant radiotherapy and/or doxorubicin-based chemotherapy.[19] While this is a mainstay of treatment, there is no consensus on the optimal treatment strategy. Local recurrence occurs in up to 50% of cases, usually within 2 years, although some studies have shown the 5 year local and distant recurrence rates to be 12% and 39%, respectively.[6,17] Lungs and bone are frequent sites of metastases, but regional lymph nodes can also be involved in 20% of cases.[6,17] Patients with favorable prognostic factors (calcifying variants and SSX2 involved fusions) have shown 10 year survival rates of 43–63%.[6]

Reports of SS arising from, near, or metastatic to the spine are rare and difficult to find in the journals. In this article, we present the experience at our institution with these cases and review the available literature.

## MATERIALS AND METHODS

We searched the patient and surgical pathology databases at our institution using the keywords *synovial sarcoma, spine, paraspinal, cervical, thoracic, or lumbar* and identified three patients diagnosed and treated for spinal SS since 2004. We also searched PubMed at http://www.ncbi.nlm.nih.gov/pubmed/ using the same keywords and found 14 cases reported in the available literature.

## RESULTS

### Case 1: Thoracic Dumbbell SS

A 59-year-old woman presented with a two-year history of constant, progressively worsening, left-sided upper thoracic pain. In the month prior to presentation, she also experienced ascending paresthesias from her toes to the T5 dermatome along with gait weakness and instability. Imaging studies elsewhere revealed a dumbbell-shaped upper thoracic mass emanating from the T5 foramen with extensive encasement and compression of the thoracic cord from approximately T4 through T6. Additionally, the tumor appeared to extend into the T5 vertebral body. A CT-guided biopsy of the mass was performed and was diagnosed as a “spindle cell tumor consistent with schwannoma.” Since her symptoms continued to progress despite treatment with steroids and radiation, she was referred to our institution for further treatment including surgical resection.

**Figure 1:**
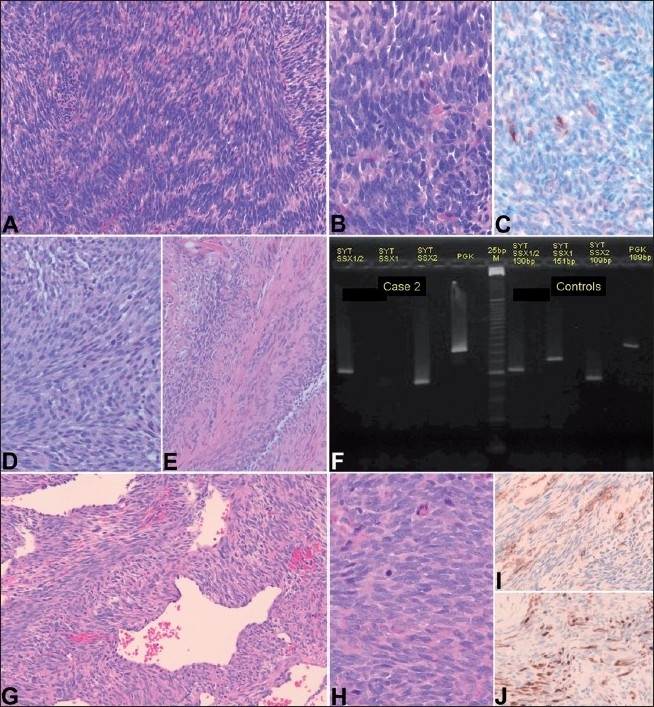
Case 1 (A–C) with low power (A) and high power (B) spindle cell appearance and focal epithelial membrane antigen immunoreactivity (C). Case 2 (D–F) illustrating the morphologic appearance of the paraspinal biopsy (D) and leptomeningeal infiltration (E). The RT-PCR (F) detects the chimeric fusion transcripts SYT-SSX2 using specific primers. Case 3 (G–J) Low power appearance (G) of the tumor with gaping vessels (so-called “hemangiopericytomatous vascular pattern”) and high power (H). The tumor expresses both epithelial membrane antigen (I) as well as cytokeratin (J) immunoreactivity

Following a lengthy discussion, the patient chose to undergo partial resection of her lesion. The tumor was exposed through a T3-T6 laminectomy and an extensive epidural mass was noted dorsally between T4 and T5 and extending out the T4-5 foramen, infiltrating the T4-5 facets and encasing the T4 nerve root. The tumor was grossly debulked, and the final pathology report revealed a high-grade SS [Figure 1]. The patient tolerated surgery well and there were no complications. Since the pathology showed a high-grade sarcoma, adjuvant therapy was planned to reduce her overall tumor burden before a second-staged surgical procedure. After she recovered from her surgery, the patient was started on three cycles of ifosfamide/adriamycin (7500 mg per sq. m/60 mg per sq. m, respectively) chemotherapy. She then returned home and received 46 Gy of total radiation to her tumor volume in addition to the previous 4 Gy received.

The adjuvant therapy had stabilized her disease and 7 months following her initial evaluation she underwent a second-staged operation. A one-piece gross total resection of T4, 5, 6, and 7 vertebra with a posterior instrumented spinal fusion from T1-L1 was performed. The tumor was removed with negative margins. The patient recovered well from the operation. She was seen on a 6-month basis by oncology with no recurrence of her tumor noted and improving symptoms. A year and a half later, she returned, complaining of new-onset back pain in her right scapular region and left mid back. This pain was found to be attributed to a fractured rod, which was surgically repaired. She continues to be followed and she has no evidence of tumor recurrence or metastases 67 months after the final resection.

### Case 2: Paraspinal SS with Leptomeningeal Spread

**Figure 2:**
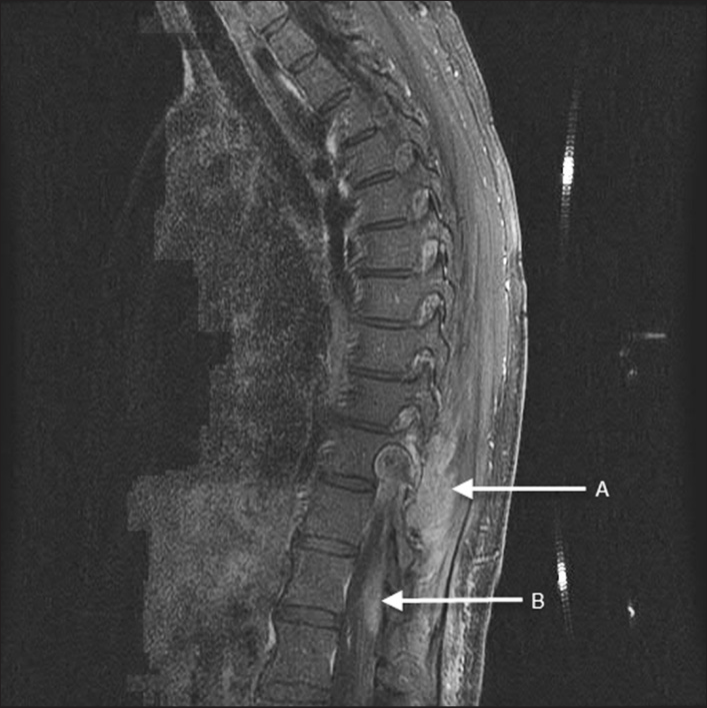
Preoperative T1-sagittal MRI with contrast shows (A) a paraspinal mass extending through the foramen as well as (B) leptomeningeal spread

A 54-year-old woman presented with several months of pain in her right buttock and hip, paresthesias of her right leg, and generalized right leg weakness. Her symptoms progressed, involving her left leg in a similar fashion and a sensory level at the costal margin was found bilaterally. Over the last month, she noted saddle numbness and loss of bladder and bowel control. Spine MR showed a large, right-sided, paraspinal mass centered around T10 with adjacent leptomeningeal enhancement—no spinal cord compression was noted [Figure 2]. A CT-guided biopsy was consistent with a spindle cell tumor, but the specific tumor type was indeterminate.

There was some question if the leptomeningeal enhancement was associated with the paraspinal mass or was an unrelated disease process. The location of maximum leptomeningeal enhancement was at the T12-L1 junction. A T12-L1 laminectomy was performed and the underlying thickened arachnoid was biopsied. The leptomeninges demonstrated the presence of a spindle cell tumor similar in morphology to the one seen in the epidural CT-guided biopsy. The tumor cell morphology suggested the diagnosis of monophasic SS, a diagnosis confirmed by immunohistochemical and RT-PCR studies [Figure 1]. The tumor arose from a paraspinal mass and extended through the dura to the leptomeninges, a very unusual presentation for SS. There were no metastatic findings at that time and systemic chemotherapy (ifosfamide and doxorubicin) was recommended. The patient returned home to receive treatment at another institution. We learned through correspondence with her home institution that the patient died 4 months later most likely from a right-midbrain lesion that appeared consistent with a glioma on MR imaging studies. No autopsy was performed.

### Case 3: T5-6 Paraspinal SS

**Figure 3:**
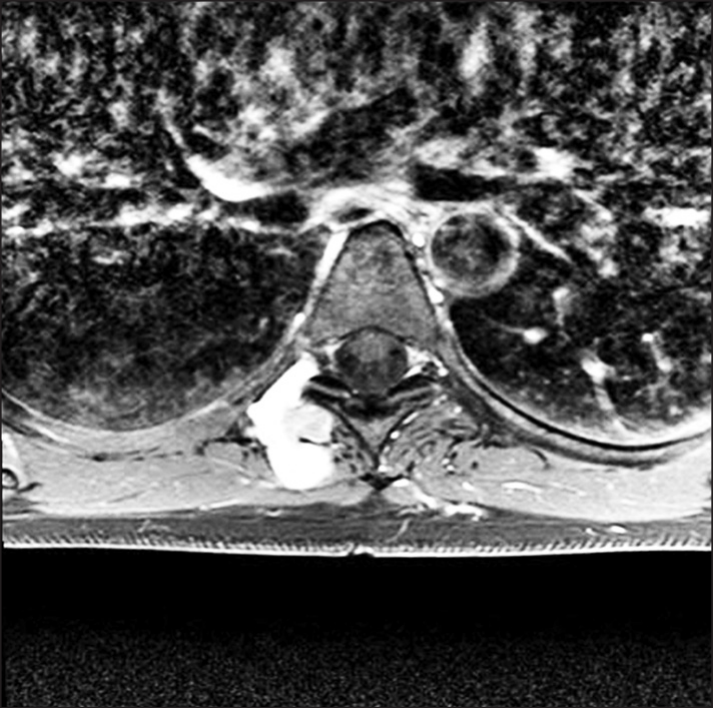
Preoperative T1-axial MRI with contrast shows an enhancing paraspinal mass with foraminal extension

This 32-year-old woman presented with 8 years of progressive right-sided subscapular thoracic pain incited by lifting her arm above her head. This pain became more frequent and began to occur spontaneously, disrupting her sleep and causing her significant difficulties. Two MRI studies were performed during this time, the first in 2004 and the second in 2009, and she was told that the findings were unremarkable. When she presented to our institution, she had severe, mechanically reproducible pain located several inches to the right of her mid-thoracic spine. The pain also radiated both up to the axilla as well as down the right leg. A review of outside MRI studies revealed a lobulated, T1 isointense, mildly T2 hyperintense, 3 cm enhancing mass involving the right T5 nerve root through the foramen with extension into the paraspinal muscles [Figure 3]. These imaging characteristics were most consistent with a schwannoma or another nerve sheath tumor since there was little change on interval exams that were 5 years apart.

As it was felt that this symptomatic lesion was likely benign and easily accessible, resection was advised. Intraoperatively, the mass was identified in the erector spinae muscles of the T4-7 region on the right side. The tumor was mobilized and gross total resection achieved, including the foraminal component. However, intraoperative pathology revealed a spindle cell sarcoma rather than a benign process, necessitating extension of the surgical resection. A T5-6 laminectomy was then performed, the dura incised, and the entire nerve root in the foramen of presumed origin was resected until negative dural margins were achieved. After the dural closure, all grossly visible tumor appeared to have been removed from the region and surrounding tissues. Final pathology showed a high-grade, fibrous-type, monophasic SS. The patient tolerated the surgery well and was neurologically intact.

Concern for residual tumor remained as postoperative imaging studies demonstrated abnormal T5-6 transverse processes. To achieve a complete resection with negative margins a second surgery was undertaken where a one-piece gross total resection was performed of the entire tumor bed. Both transverse processes were resected and gross residual tumor was found in the T6 transverse process. Wide margins were excised, and the pathologist entered the OR to correlate the negative margins with the visualization of the previous tumor bed. Based on these findings, it appeared that the resection had removed all residual viable tumor present. The patient tolerated the second operation well.

At 6-month follow-up, no local tumor recurrence was found, but several small lung nodules were noted on chest CT. These nodules were consistent with metastases and the largest was measured at 7 mm. She was seen in oncology and systemic chemotherapy to shrink the tumors and resection of these nodules has been planned. At 1-year follow-up, her spine continues to be disease free, while her lung nodules have slightly increased in size. Therapy is ongoing at this time to address the lung nodules.

## DISCUSSION

SS is a rare, aggressive neoplasm of uncertain origin predominantly affecting adolescents and young adults.[6,30] It is associated with a balanced reciprocal translocation t(X:18)(p11:q11). SS presents in a variety of histopathological forms, from monophasic, being uniformly comprised of spindle cells, to biphasic, with epithelial and spindle cell components.[6,9,14,25,30,33] Due to histological variety, SS can be mistaken for numerous other mesenchymal or nonmesenchymal tumors, making immunohistochemical and molecular studies important in achieving the correct diagnosis.[13]

Definitive characteristics making SS completely distinguishable on radiologic examination have not been seen, making diagnosis difficult.[20] Plain radiographs may be normal in up to 50% of patients with SS, making it difficult to visualize unless the tumor is eroding adjacent bony structures. However, tumor calcification may be seen in approximately 30% of patients and these calcifications become readily apparent on routine radiography or computerized tomography.[29] On CT imaging, SS may appear as a well demarcated, hypodense mass with homogenously or heterogeneously enhancement, making it easy to confuse with other benign or malignant tumors.[20] MRI has proven to be the superior modality for detecting SS. In cases localized to the head and neck, it has been shown that these tumors possess a signal intensity similar to fat on T2-weighted images, and isointense as compared to gray matter on T1-weighted images.[11]

It is widely accepted that the current most effective treatment for SS is a wide surgical excision with negative margins.[6,13,19] Use of adjuvant radiation therapy decreases the local recurrence rate.[1] Radiation proved to be superior to chemotherapy alone as adjuvant therapy to primary surgical excision.[10] A single chemotherapy protocol has not yet been proven most effective in treating SS, but two agents, doxorubicin and ifosfamide have demonstrated meaningful activity in the treatment of soft tissue sarcomas.[19] Specifically, high dose ifosfamide has been associated with improved disease-specific survival in adult patients with high-risk primary SS and should be considered a standard part of the chemotherapy regimen for this disease.[5,23]

Most of the available information on SS has come from tumors localized to the extremities. However, up to 5% of these tumors are encountered in the body axis, including the spine, mediastinum, retroperitoneum, and head and neck region. Reports of SS located within the spinal axis have been rare and are limited to 13 case reports on 14 patients published in the literature [Table 1]. Of these 14 patients with spinal SS, 8 were within the paraspinal musculature (with most having foraminal extension), 3 were intradural and associated with spinal nerve roots, 2 were metastatic lesions (one to bone, the other intramedullary), and 1 was a bony/lytic lesion. The cases we present here all involved the paraspinal muscles with two of them being associated with spinal nerve roots, the other having significant leptomeningeal extension, this finding not previously reported in the literature.

**Table 1:** Review of spinal SS at our institution and reported cases in the literature by year

Our institution	Age	Imaging	Treatment	Outcome
Case 1: Thoracic dumbbell**SS**	59 F	Imaging studies revealed a dumbbell-shaped upper thoracic mass emanating from the T5 foramen with extensive encasement and compression of the thoracic cord from approximately T4 through T6. Additionally, the tumor appeared to extend into the T5 vertebral body.	Two stage operation performed. First, a T3-5 laminectomy with debulking of tumor was performed. Three cycles of ifosfamide/ adriamycin (7500 mg per sq. m/60 mg per sq. m, respectively) chemotherapy performed. The patient received 46Gy of total radiation to tumor volume. The second operation consisted of en bloc resection of T4, 5, 6 and 7 vertebra with a posterior instrumented spinal fusion from T1-L1. The tumor was removed to negative margins.	Patient continues to be followed and has no evidence of tumor recurrence or metastases at 67 months from the final resection.
Case 2: Paraspinal**SS**with leptomeningeal spread	54 F	Spine MRI showed a large, right-sided, paraspinal mass centered around T10 with adjacent leptomeningeal enhancement—no spinal cord compression was noted. CT-guided biopsy findings were consistent with a spindle cell tumor.	The tumor cell morphology suggested the diagnosis of monophasic**SS, **a diagnosis confirmed by immunohistochemical and RT-PCR studies. The tumor was arising as a paraspinal mass and extending through the dura to the leptomeninges. There were no metastatic findings at that time and systemic chemotherapy (Ifosfamide and Doxorubicin) was recommended.	The patient returned home to receive care at another institution. The patient died 4 months later.
Case 3: T5-6 paraspinal**SS**	32 F	MRI studies revealed a lobulated, T1 isointense, mildly T2 hyperintense, enhancing mass involving the right T5 nerve root through the foramen with extension into the paraspinal muscles. The mass measured 3.1 × 2.0 × 2.5 cm^3^ and there was no extension into the spinal canal.	Intraoperatively, the mass was identified in the erector spinae muscles of the T4-7 region on the right side. The tumor was mobilized and removed. A T5-6 laminectomy was performed, the dura was incised and the entire nerve root in the foramen of presumed origin was resected including the dural attachments until the dural margins were negative. A second operation was performed to resect gross residual tumor found in the T6 transverse process.	At six-month follow-up, no tumor recurrence was found, but several small lung nodules were noted on chest CT. These nodules were consistent with metastases and the largest was measured at 7 mm. Systemic chemotherapy to shrink the tumors and resection of these nodules was been planned after her most recent visit.

**Literature cases by year**	**Age**	**Imaging**	**Treatment**	**Outcome**

Arnold*et al.,*2010[2]	26 F	CT showed destruction of odontoid and C2 body. MRI revealed tumor in posterior C2, ventral epidural space from C2-5 with narrowing of spinal canal. Widely metastatic disease confirmed.	Spinal cord decompression with C2-3 laminectomy and posterior C2 corpectomy. Occipital-C7 fusion. Scheduled for palliative chemotherapy.	Developed fever, leukocytosis and acidosis, leading to sepsis. Multiple metastases to liver and abdomen. Patient died 6 months later
Barus*et al.,*2009[3]	14 F	MRI showed a large lesion in lumbar paraspinal muscles from L2 to sacrum with extension into spinal canal and L3-4 thecal sac.	Initial biopsy showed SS. Marginal resection to preserve neural elements performed. A 310 g lobulated mass removed with fibrous pseudocapsule. Two cycles of ifosfamide/ doxorubicin chemotherapy and total dose of 5940 cGy administered followed by four cycles of chemotherapy	Patient developed chronic kidney disease. Almost 6 years postop., patient is free of local recurrence or metastatic disease.
Koehler*et al.,*2009[15]	60 M	MRI showed large right-sided paraspinal mass from T7-9. Calcifications and enhanced signal intensity of the central tumor segment.	Right-sided thoracotomy and biopsy of tumor mass performed. Upon malignant confirmation, wide resection with negative margins performed. Vascular and neuro supply ligated and resected. Radiation therapy given.	9 month postoperative imaging showed no recurrence.
Ravnik*et al.,*2009[21]	32 M	Myelography showed a T12/L1 Contrast Block. CT/MRI showed intraspinal epidural T12-L2 tumor.	Immediate surgical decompression. Three level laminectomy with epidural mass removal. Second debulking surgery. Six cycles (ifosfamide/doxorubicin) and 50.4 Gy of radiation.	Local recurrence after 12 months. Further treatment refused.

**Literature cases by year**	**Age**	**Imaging**	**Treatment**	**Outcome**

Scollato*et al.,*2008[27]	59 M	MRI showed intramedullary lesion at C3-5. Right pulmonary mass seen.	Surgery performed after chemotherapy was refused by patient. Longitudinal myelotomy performed.	Patient experienced postoperative pain relief but no neurological change. Patient died of lung and hepatic metastases 3 months later.
de Ribaupierre*et al.,*2007[22]	11 F	MRI showed intradural, heterogeneous mass in right C6-7 foramen.	Right C6-7 foraminotomy with complete resection. Nerve roots found to be involved and were resected. Postop. chemotherapy administered (vincristine, actinomycine, and ifosfamide) as well as local radiotherapy.	Disease free for 16 months then local reoccurrence found. Another operation performed and further chemotherapy/radiation given. Patient died 6 years after diagnosis.
Greene*et al.,*2006[8]	11 F	MRI showed a large intradural, extramedullary lesion at L2-4, filling canal and displacing nerve roots laterally. Multiple other nodules at C6, T2, T5, T8 and L1 levels noted	Large intradural, extramedullary lesion from L2-4 seen. No dural involvement. Near total resection, small capsule remnant. Four courses of doxorubicin/ifosfamide delivered. Radiation given, 45 Gy to total spine, 54 Gy boost to lumbosacral region.	Five months after diagnosis, MRI showed four intracranial metastases. Intracranial resection and radiation performed. Patient died 14 months later.
Sakellaridis*et al.,*2006[24]	36 F	MRI showed recurrent tumor of lumbar spine attached to dura at L2-3. Previous diagnosis of hemangiopericytoma.	Extended laminectomy L1-3 with resection of tumor mass. Dura was opened and subarachnoid invasion was noted. 3500 rads given.	2 years postop. new C7 lesion and mediastinal tumor from T5-10. Third operation performed. Patient died 1.5 year later from metastases.
Suh*et al.,*2005[29]	44 M	MRI demonstrated large paravertebral and epidural mass displacing thecal sac at L4-5. No distant metastases seen.	Hemilaminectomy/facetectomy at L4-5 performed. Mass was found to extend through right L4 foramen. Near-total resection performed. Patient refused chemotherapy. Radiation delivered to residual mass.	At time of report, patient’s symptoms showed improvement. No long-term follow-up.
Morrison*et al.,*2001[18]	53 F	MRI showed a large paraspinal mass from C7-T3 without dural or foraminal involvement. No metastases seen.	Needle biopsy showed a spindle cell tumor. Complete surgical resection achieved.	No outcome or follow-up reported.
Wu*et al.,*2000[32]	30 M	MRI showed an enhancing mass in the right paraspinal muscles from T12-L1 with extension into the spinal canal and displacement of the thecal sac. CT showed bony erosion of the pedicles, transverse process and posterior elements of the affected levels	Needle biopsy showed a myxoid mesenchymal tumor. Immunohistochemistry confirmed SS with an open biopsy. No resection was reported	No outcome or follow-up reported.
Signorini*et al.,*1986[28]	59 M	CT showed a lytic lesion involving the T2 vertebral body with right pedicle erosion. A myelogram showed a complete block at that level.	Upfront radiation—48 Gy. 6 months later developed acute paraplegia. Underwent right transpleural subtotal resection of the T2 vertebral body. Pathology showed a biphasic SS	Died 3 months later from disease progression.
Treu*et al.,*1986[31]	21 M	Patient 1 had a CT which showed a mass eroding the posterior arch of C1 with some dural compression.	Patient 1 had a subtotal resection through a posterior approach. A monophasic SS was identified.	Patient 1: 25 months later developed metastates to pancreas—no follow-up thereafter reported.
	18 M	Patient 2 had a CT scan which showed a lumbar paraspinal mass from L4-L5.	Patient 2 had a gross total resection where the soft tissue component, transverse process of L4 and 5 and part of the psoas muscle was resected. Pathology confirmed a monophasic SS.	Patient 2: No outcome or follow-up reported.

Clinical differential diagnosis for SS involving the spine includes primarily nerve sheath tumors, and most SS are assumed to be benign nerve sheath tumors preoperatively. Indeed, in two of our cases a simpler/smaller operation was performed first because the lesions were thought to be benign, however, when the final pathology identified SS, a larger more radical procedure was performed. With this limited data, the long-term outcomes are hard to quantitate for SS involving the spine. Based on the previous case reports, most patients died within 3 years following diagnosis, with the exception of a 14-year-old girl who remained disease-free at 6 years.[3]

Over half of the reported spinal SS had dumbbell-shaped intraforaminal extension with a larger extraspinal and smaller intraspinal component. In most cases, the patients’ symptoms were caused by the intraspinal component and resultant compression of neural elements. It was striking however, how large the extraspinal portion was in many of the cases. The most common tumor presenting in this fashion is a benign schwannoma, whereas, malignant nerve sheath tumors are very rare. In regards to SS, it is the rapid growth of the extraspinal portion that is most suggestive of a malignant process. However, in one of our cases, the patient had serial MRI scans 5 years apart and there was minimal growth during this time, which is most unusual for a sarcoma.

Some of these unique properties of SS are just now being understood at a molecular level. It has been recently reported that microRNAs (miRNAs) may play an expanded role in the tumorigenesis of some cancers, and when deregulated, depending on their mRNA targets, they can act to down regulate tumor suppressor genes and give a growth advantage to tumor cell lines.[4] In SS, a unique pattern of deregulated miRNAs has been found that is distinct from muscle tissue and a wide range of other sarcoma types.[12] Specifically, the overexpression of a microRNA, miR-183, has been found to act as an oncogene through down regulation of EGR1 translation, a tumor suppressor that is correlated strongly with tumor formation and transformation processes when its levels are depleted.[26] Another overexpressed miRNA, let-7e, has been shown to down regulate expression of HMGA2, a transcription factor which works in concert with the SS18-SSX fusion product to decrease levels of SNAI1, a transcriptional repressor, ultimately causing epithelial differentiation and transition in SS.[12] Importantly, when let-7e was inhibited by a miRNA inhibitor, the proliferation of the SS cells were suppressed. While these findings are very new, they provide a better understanding of the underlying molecular basis for SS tumorigenesis and also present possible future targets for pharmacological therapies.

Experts agree that the cornerstone of treatment for SS is wide surgical excisions with negative margins. Based on our experience, if a spinal dumbbell tumor has any characteristic that would be unusual for a benign nerve sheath tumor, a needle-guided biopsy should be performed first to obtain a diagnosis. Ideally, this is followed by a definitive operation to try and remove the tumor en bloc with negative margins. Traditionally, a 5 cm margin defines a negative margin in sarcoma surgery, however, in many of these cases this would encompass critical structures such as the spinal cord. Thus, in many cases, the best that can be hoped for is a gross total resection with “marginal margins” in which there is no pathologic specimen, but critical structures are not injured.

## CONCLUSION

SS of the spine can be challenging to diagnose and even harder to treat. Best available evidence suggests that a multimodal treatment strategy starting with aggressive surgical resection followed by radiation and chemotherapy offers the patients the best chance for a cure. The underlying molecular genetics for SS tumorigenesis is just now being elucidated and hopefully will lead to a better understanding in regards to tumor pathology and lead to novel therapeutics in the future.
